# Transmission Dynamics and Short-Term Forecasts of COVID-19: Nepal 2020/2021

**DOI:** 10.3390/epidemiologia2040043

**Published:** 2021-12-16

**Authors:** Sushma Dahal, Ruiyan Luo, Raj Kumar Subedi, Meghnath Dhimal, Gerardo Chowell

**Affiliations:** 1 Department of Population Health Sciences, School of Public Health, Georgia State University, Atlanta, GA 30303, USA; rluo@gsu.edu (R.L.); gchowell@gsu.edu (G.C.); 2 Bhaskar Tejshree Memorial Foundation, Kathmandu 44600, Nepal; rajkumarsubedi@gmail.com; 3 Nepal Health Research Council, Kathmandu 44600, Nepal; meghdhimal@gmail.com

**Keywords:** transmission dynamics, COVID-19, short-term forecast, Nepal, 2020/2021, pandemic

## Abstract

Nepal was hard hit by a second wave of COVID-19 from April–May 2021. We investigated the transmission dynamics of COVID-19 at the national and provincial levels by using data on laboratory-confirmed RT-PCR positive cases from the official national situation reports. We performed 8 week-to-week sequential forecasts of 10-days and 20-days at national level using three dynamic phenomenological growth models from 5 March 2021–22 May 2021. We also estimated effective and instantaneous reproduction numbers at national and provincial levels using established methods and evaluated the mobility trends using Google’s mobility data. Our forecast estimates indicated a declining trend of COVID-19 cases in Nepal as of June 2021. Sub-epidemic and Richards models provided reasonable short-term projections of COVID-19 cases based on standard performance metrics. There was a linear pattern in the trajectory of COVID-19 incidence during the first wave (deceleration of growth parameter (*p*) = 0.41–0.43, reproduction number ($R_{t}$) at 1.1 (95% CI: 1.1, 1.2)), and a sub-exponential growth pattern in the second wave (*p* = 0.61 (95% CI: 0.58, 0.64)) and $R_{t}$ at 1.3 (95% CI: 1.3, 1.3)). Across provinces, $R_{t}$ ranged from 1.2 to 1.5 during the early growth phase of the second wave. The instantaneous $R_{t}$ fluctuated around 1.0 since January 2021 indicating well sustained transmission. The peak in mobility across different areas coincided with an increasing incidence trend of COVID-19. In conclusion, we found that the sub-epidemic and Richards models yielded reasonable short-terms projections of the COVID-19 trajectory in Nepal, which are useful for healthcare utilization planning.

## 1. Introduction

South Asia is the most populated region of the world, with a population of about 2 billion. This region includes eight of the world’s 36 megacities with an urban population greater than 10 million and population density greater than 10,000 per square kilometer [1]. When the first confirmed case of COVID-19 in the region was reported in Nepal, the public health community feared for the worst given the high poverty and population density levels and a poorly resourced health care system [2]. Nevertheless, South Asia was not significantly affected by the first COVID-19 wave in 2020 compared to many developed countries. Yet, the situation took a different turn when neighboring India reported the emergence of the Delta variant (also called B.1.617) on 5 October 2020 [3], the most transmissible variant of COVID-19 reported as of September 2021 [3,4,5]. Since early March 2021, the Delta variant started to take hold of the Indian population and fueled a devastating second wave that engulfed South Asia. As of 30 November 2021, South Asia reported a total of 47.9 million confirmed cases (18.91% of global total) and 767,195 COVID-19 deaths (15.04% of global total) [6].

Nepal is one of the South Asian countries most heavily affected by the COVID-19 pandemic. By 30 November 2021, the Himalayan country of roughly 30 million people had reported 821,366 RT-PCR positive cases and 11,526 COVID-19 deaths, out of a total of 4.6 million RT-PCR tests performed in the country covering approximately 15 percent of the total population [7]. The impact of the COVID-19 pandemic is especially detrimental for countries such as Nepal, where the health infrastructure is fragile and less equipped. In Nepal, there are only 194 hospitals with ICU facilities, with a capacity of 26,930 hospital beds, 3076 isolation beds, 1595 ICU beds, and 840 ventilators [8]. The physician per 100,000 population ratio is only 0.7, and to manage the shortage of health workers during the pandemic, the Nepal government had to call back those health workers on long-term leave [9].

Nepal shares a border of 1125 miles with India and has historically allowed free movement of people between the two countries for tourism, education, healthcare, marriage, and economy [10]. Although the movement across these borders was restricted during the initial period of the COVID-19 pandemic, the movement across borders resumed in early 2021 [11]. As the devastating second wave started in India, tens of thousands of Nepalese migrants living in India often returned to Nepal through porous borders without undergoing testing or quarantine protocols [12]. In addition, instances of crowded movement of people in the country, lack of practice of social distancing guidelines [13], and the start of the Nepalese New Year and wedding season (from 23 April 2021), elevated the reproduction number [14], giving rise to the second wave of COVID-19 in Nepal during April–June 2021. Moreover, the political crisis that was ongoing in the country after the prime minister dissolved parliament in December 2020, followed by pro-and anti-protests and rallies in the country, and neglect by the government in preparing for the second wave hindered the effective planning and response efforts [15,16]. During the second wave, Nepal’s under-resourced public health system was already overstretched beyond capacity, and many COVID-19 patients unnecessarily died due to a lack of oxygen supply [17].

From late July 2021, Nepal again saw a mild resurgence wave of COVID-19, which occurred after a few weeks of steady decline in incidence during the second wave. The low testing rates, weak social distancing guidelines, an open border policy with India, and poor economic conditions likely negatively affected the prevalence of COVID-19 in Nepal. Mathematical models help shed light on past and present outbreaks as well as predict the future trends [18]. In this paper, we investigate the transmission dynamics of COVID-19 in Nepal by analyzing case incidence data at the national and provincial levels. Understanding the dynamics of disease transmission and the potential role of mitigation strategies at the national and provincial level can help guide control efforts.

To forecast the epidemic trajectory, we utilize established and validated mathematical models previously used to forecast and investigate dynamics of SARS, MERS, Ebola, and the COVID-19 pandemic in other settings [19,20]. We estimate the effective and instantaneous reproduction numbers of SARS-CoV-2 at the regional and national levels to understand the transmission dynamics of the virus and examine the mobility trends in relation to the implementation of lockdowns.

## 2. Materials and Methods

### 2.1. Setting

The Federal Democratic Republic of Nepal is a landlocked country located between India and China. The country is divided into 7 provinces, which are further divided into districts. There are total 14 districts in province 1, 8 in province 2, 13 in Bagmati province, 11 in Gandaki province, 12 in Lumbini province, 10 in Karnali province, and 9 in Sudurpaschim province [21].

Nepal confirmed its first case of COVID-19 on 23 January 2020, in a 32-year-old male Nepalese student based at Wuhan University of Technology who had returned to Nepal. The second case was detected after two months on 23 March 2020. By 31 May, a total of 1572 cases and eight deaths had been reported in Nepal [8]. After the confirmation of the second case, the government imposed a national lockdown on 24 March and sealed its borders [22] until 21 July 2020 [13].

### 2.2. Data

For short-term forecasting of the COVID-19 epidemic in Nepal, and for estimating reproduction number at the national and provincial level from the case incidence data, we utilized publicly available time series of laboratory-confirmed RT-PCR positive cases by dates of reporting that were obtained from the COVID-19 situation reports published by Ministry of Health and Population of the government of Nepal as of 30 November 2021 [23]. Similarly, to assess the mobility trend during the course of the pandemic in Nepal, we used Google’s mobility data for Nepal [24]. Google’s mobility data shows how visits to places, such as the grocery stores, parks, and recreation spots, are changing in each geographic region. A baseline day represents the normal value for that day of the week. The baseline day is the median value from the 5-week period from 3 January–6 February 2020. In this study, we analyzed Google’s mobility data from 15 February 2020 to 30 November 2021.

### 2.3. Modeling Framework for Forecast Generation

We utilized three dynamic phenomenological growth models to generate short-term forecasts for Nepal (10-days and 20-days ahead). These models have been applied to various infectious diseases including SARS, foot and mouth disease, Ebola [25], and the current COVID-19 outbreak [26,27]. Phenomenological growth models applied in this study include the following differential equation models: the generalized logistic growth model [25], the Richards growth model [28], and the sub-epidemic model [29]. The forecasts obtained from these dynamic growth models can provide an assessment of the potential scope of the outbreak in near real-time and insight on the impact of the implementation and relaxation of control interventions, which help guide public health policy. The description of these models is provided as follows.

#### 2.3.1. Generalized Logistic Growth Model

The generalized logistic growth model (GLM) [25] displays a range of epidemic growth profiles, including the polynomial and exponential growth patterns. GLM characterizes epidemic growth by estimating three parameters: (i) the intrinsic growth rate, *r* (ii) a dimensionless “deceleration of growth” parameter, *p* and (iii) $k_{0}$, the epidemic size. The varied epidemic growth patterns are observed by the modulation of the deceleration of growth parameter resulting in the exponential growth dynamics (*p* = 1), the sub-exponential growth (0 < *p* < 1), or the constant incidence (*p* = 0) patterns. The GLM model is given by the following differential equation:

$$\frac{dC\left(t\right)}{dt}=rC{\left(t\right)}^{p}\;\left(1-\frac{C\left(t\right)}{k_{0}}\right),$$

where *C*(*t*) denotes the cumulative number of cases over time *t*, and $\frac{dC\left(t\right)}{dt}$ describes the incidence curve over time *t*.

#### 2.3.2. Richards Growth Model

The Richards model [28] extends the simple logistic growth model by incorporating a scaling parameter, a, that measures the deviation from the symmetric simple logistic growth curve [28,30,31]. The Richards model is given by the differential equation:

$$\frac{dC\left(t\right)}{dt}=rC\left(t\right)\left[1-{\left(\frac{C\left(t\right)}{k_{0}}\right)}^{a}\right],$$

where *C*(*t*) represents the cumulative case count at time *t*, *r* is the growth rate, *a* is a scaling parameter, and $k_{0}$ is the final epidemic size.

#### 2.3.3. Sub-Epidemic Model

The sub-epidemic model [29] can be used to model an epidemic wave comprising of multiple overlapping sub-epidemics where each sub-epidemic is modeled using a GLM model denoted by the following differential equation:

$$\frac{dC\left(t\right)}{dt}=rC^{p}\left(t\right)\left(1-\frac{C\left(t\right)}{k_{o}}\right),\;$$

We model an epidemic wave comprising of *n* overlapping sub-epidemics using a system of coupled differential equations, as follows,

$$\frac{dC_{i}\left(t\right)}{dt}=rA_{i-1}\left(t\right)C_{i}{\left(t\right)}^{p}\left(1-\frac{C_{i}\left(t\right)}{k_{i}}\right),$$

where $C_{i}\left(t\right)$ is the cumulative cases for the ith sub-epidemic, and $k_{i}$ is the size of ith sub-epidemic where *i* = 1…*n*. Hence when *n* = 1, the sub-epidemic model reduces to the simple logistic-type model. To model the onset timing of the (*i* + 1)th sub-epidemic, we use an indicator variable $A_{i}\left(t\right)$, making sure that the sub-epidemics comprising an epidemic wave follow a regular structure. Therefore,

$$A_{i}\left(t\right)=\{\begin{array}{c} 1\;\mathrm{if}\;C_{i}\left(t\right)>C_{thr}\\ 0\;\mathrm{Otherwise} \end{array}\;\mathrm{for}\;i=1,2,3,\ldots n\;,$$

where $1\leq C_{thr}<k_{o}$ and $A_{1}\left(t\right)=1$ for the sub-epidemic 1. For the subsequent sub-epidemics, the size of the ith sub-epidemic ($k_{i}$) declines exponentially at a rate *q*. This can happen due to multiple factors such as the seasonal transmission effect, effect of interventions and population behavior changes. If *q* = 0, then the sub-epidemic model predicts an epidemic wave composed of equal sized sub-epidemics [29]. If we assume that the subsequent sub-epidemic sizes decline exponentially, we get $k_{i}=k_{0}e^{-q\left(i-1\right)}$, where $k_{0}$ is the size of initial sub-epidemic i.e., $k_{1}=k_{0}$. Therefore, when *q* > 0, the total number of sub-epidemics supported by the model depends on $C_{thr}$, *q* and $k_{0}$ because the (*i* + 1)th sub-epidemic is triggered only if $C_{thr}>C_{i}\left(t\right)$ [29].

### 2.4. Model Calibration and Forecasting Approach

We conducted 8 week-to-week subsequential 10-days and 20-days ahead short-term forecasts at the national level utilizing the data retrieved from Ministry of Health and Population, Government of Nepal by the dates of report [23]. Table 1 shows the calibration and forecast period for each of the three dynamic models used.

For each of the models, we estimated the best fit solution using non-linear least square fitting procedure [32]. This process minimizes the sum of squared errors between the model fit $f(t,\hat{\Theta }$) and the smoothed data estimates $y_{t}$, and yields the best set of parameter estimates $\Theta =\left(\theta_{1},\theta_{2},\ldots ,\theta_{m}\right)$, where the smoothed data was obtained by using smoothfactor = 7 in Matlab. The smooth function in Matlab reduces the noise within a data set by using a moving average method. Let $\hat{\Theta }$ = argmin $\sum_{t=1}^{n}\left(f(t,\hat{\Theta }\right)-y_{t}{)}^{2}$ denote the best fit estimates. Here $\hat{\Theta }=\left(r,p,k_{o}\right)$ corresponds to the set of estimates for the parameters of the GLM model $\hat{\Theta }=\left(r,k_{o},a\right)$ corresponds to estimates of parameters of the Richards model, and $\hat{\Theta }=\left(r,p,k_{o},q,C_{thr}\right)$ corresponds to the estimates of parameters of the sub-epidemic model [30]. While for the sub-epidemic model, we provided the initial best guesses of the parameter estimates, for GLM and Richards growth model, we initialized the parameters for the nonlinear least squares’ method [32] over a wide range of plausible parameters sampled from a uniform distribution. This allowed us to test the uniqueness of the best fit solution. Moreover, the initial conditions were set at the first data point for both models [30]. Uncertainty bounds around the best-fit solution were generated using parametric bootstrap approach assuming a negative binomial error structure for each of the models, where the variance was calculated by averaging mean to variance ratio in the data. The details of this method are provided in ref [30].

For each model, we generated M = 300 datasets by the bootstrap approach during the calibration phase, refitted the model to each generated dataset, and used the M sets of parameters estimates to construct the 95% confidence intervals for each parameter. Further, each M best fitted model was used to generate m = 30 additional data points with negative binomial error structure extended through the forecasting period. For the forecasting period, we constructed the 95% prediction intervals with these 9000 (M × m) curves. Detailed description of the methods of parameter estimation can be found in references [30,33,34].

### 2.5. Performance Metrics

We utilized the following five performance metrics to assess the quality of our model fit and the 10-days and 20-days ahead short-term forecasts: root mean squared error (RMSE) [35], the mean absolute error (MAE) [36], the mean interval score (MIS) [35], the coverage of the 95% prediction intervals [35], and the weighted interval score (WIS) [37] for each of the three models. For calibration performance, we compared the model fit to the smoothed incidence data fitted to the model, whereas for the performance of forecasts, we compared our forecasts with the incidence data for the time-period of the forecast.

The root mean squared error (RMSE) and the mean absolute error (MAE) assess the average deviations of the model fit to the observed data in $L_{2}$ and $L_{2}$ norm, respectively. The root mean squared error (RMSE) is given by

$$\mathrm{RMSE}=\sqrt{\frac{1}{n}\overset{n}{\underset{i=1}{\sum }}{\left(f\left(t_{i},\hat{\Theta }\right)-y_{t_{i}}\right)}^{2},}\;$$

and the mean absolute error (MAE) is given by

$$\mathrm{MAE}=\frac{1}{n}\overset{n}{\underset{i=1}{\sum }}\left|f\left(t_{i},\hat{\Theta }\right)-y_{t_{i}}\right|,$$

where $y_{t_{i}}$ is the time series of cases by date of onset, $t_{i}$ is the time stamp, and $\hat{\Theta }$ is the set of estimated parameters. For the calibration period, *n* equals the number of data points used for calibration, and for the forecasting period, *n* = 10 and 20 for the 10-day and 20-day ahead short-term forecast respectively.

Moreover, to assess the model uncertainty and performance of prediction interval, we used the 95% PI and MIS. The prediction coverage is defined as the proportion of observations that fall within 95% prediction interval and is calculated as

$$\mathrm{PI}\;\mathrm{coverage}=\frac{1}{n}\overset{n}{\underset{i=1}{\sum }}I\left\{y_{t_{i}}>L_{t_{i}}\;\cap \;y_{t_{i}}<U_{t_{i}}\right\},$$

where $y_{t_{i}}$ are the case incidence data, $L_{t_{i}}$ and $U_{t_{i}}$ are the lower and upper bounds of the 95% prediction intervals, respectively, n is the length of the period, and I is an indicator variable that equals 1 if value of $Y_{t_{i}}$ is in the specified interval and 0 otherwise.

The mean interval score addresses the width of the prediction interval as well as the coverage. The mean interval score (MIS) is given by

$$\mathrm{MIS}=\frac{1}{n}\overset{n}{\underset{i=1}{\sum }}\left(U_{t_{i}}-L_{t_{i}}\right)+\frac{2}{0.05}\left(L_{t_{i}}-y_{t_{i}}\right)I\{y_{t_{i}}<L_{t_{i}}\}+\frac{2}{0.05}\;\left(y_{t_{i}}-U_{t_{i}}\right)I\{y_{t_{i}}>U_{t_{i}}\}.$$

In this equation, $L_{t_{i}}$, $U_{t_{i}}$, *n*, and I are as specified above for PI coverage. Therefore, if the PI coverage is 1, the MIS is the average width of the interval across each time point. For two models that have an equivalent PI coverage, a lower value of MIS indicates narrower intervals.

We also used the weighted interval score (WIS) [37,38] which is a distance sensitive score to assess the performance of model calibration and forecast. The weighted interval score (WIS) is given by

(1) $${\mathrm{WIS}}_{\alpha_{0:K}}\left(F,\;y\right)=1/\left(K+1/2\right)*\left(\omega_{0}*\left|y-m\right|+\overset{K}{\underset{k=1}{\sum }}\left\{\omega_{k}*\;IS_{\alpha_{k}}\left(F,y\right)\right\}\right)$$

where $\omega_{k}=\frac{\alpha_{k}}{2}\;\mathrm{for}\;k=1,\ldots \ldots K\;\mathrm{and}\;\omega_{0}=1/2$. Here, we use *K* = 11 interval scores for α = 0.02, 0.05, 0.1, 0.2,…0.9, *F* denotes the forecasts, and $IS_{\alpha_{k}}\left(F,y\right)=[(U_{t}-L_{t})+\frac{2}{\alpha }*\left(L_{t}-y_{t}\right)*1\left(y_{t}<L_{t}\right)+\frac{2}{\alpha }*\left(y_{t}-U_{t}\right)*1\left(y_{t}>U_{t}\right)]$ is the interval score for the (1−$\alpha_{k})\;\times \;100\%$ PI.

### 2.6. Reproduction Number

Reproduction number ($R_{t}$) characterizes the average number of secondary cases generated by a primary case at calendar time *t* during an outbreak when control measures are in place [39]. $R_{t}$ is an important public health indicator of effectiveness of public health interventions during an epidemic [39]. While $R_{t}$ estimates of more than 1 indicate continuation of widespread disease transmission, $R_{t}$ less than 1 indicates that sustained disease transmission is unlikely and the outbreak is under control [40].

### 2.7. Estimating Reproduction Number ($R_{t}$) Using GGM

We estimated the effective reproduction number ($R_{t}$) for the early ascending phase of the COVID-19 epidemic in Nepal using a generalized growth model (GGM). We estimated reproduction number ($R_{t}$) at the national level for first 30 days of the phase during initial rise in cases (25 May to 23 June 2020), and first wave (1 August to 30 August 2020) of COVID-19 pandemic. For the second wave, we estimated reproduction number for the first 30 days, at both the national and at provincial level. Based on the growth of incidence curve, we estimated $R_{t}$ for the period from 12 April to 11 May 2021 for national level and for six provinces, except for Karnali province for which we took data for the period (20 April to 19 May 2021). Similarly, we also estimated $R_{t}$ for the resurgence wave after the second wave for the first 30 days period (10 July to 8 August 2021) at the national level.

We modeled the generation interval of SARS-CoV-2 assuming gamma distribution with a mean of 5.2 days and a standard deviation of 1.72 days [41]. We estimated the growth rate parameter r, and the deceleration of growth parameter *p* from the GGM. The GGM model is used to simulate the progression of local incidence cases $I_{i}$ at calendar time $t_{i}$. This is followed by the application of the discretized probability distribution of the generation interval, denoted by $\rho_{i}$, to the renewal equation to estimate the reproduction number at time $t_{i}$ [42,43,44]

$$R_{t_{i}}=\frac{I_{i}}{\sum_{j=0}^{i}(I_{i-j}+\alpha J_{i-j})\rho_{j}}$$

The factor $J_{i}$ represents the imported cases at time $t_{i}$, $I_{i}$ denotes the local case incidence at calendar time $t_{i},$ and $\rho_{j}$ represents the discretized probability distribution of generation interval. The factor α assesses the relative contribution of imported cases to secondary disease transmission. The factor α was set at 0. The numerator represents the total new cases $I_{i}$, and the denominator represents the total number of cases that contribute (as primary cases) to generating the new cases $I_{i}\;$(as secondary cases) at time $t_{i}$. Hence, $R_{t}$, represents the average number of secondary cases generated by a single case at calendar time t. The uncertainty bounds around the curve of $R_{t}$ are derived directly from the uncertainty associated with the parameter estimates (r, *p*) obtained from the GGM. We estimated $R_{t}$ for 300 simulated curves assuming a negative binomial error structure where the variance was estimated by averaging mean to variance ratio for the local case incidence [30].

### 2.8. Estimating Instantaneous Reproduction Number ($R_{t}$)

The instantaneous reproductive number is the expected number of secondary infections occurring at time *t*, divided by the number of infected individuals, each scaled by their relative infectiousness at time *t*. We estimated the instantaneous reproduction number, $R_{t}$, at the national and regional level using the case incidence date by dates of report, using the method of Cori et al. [45] in which the instantaneous $R_{t}$ is estimated as

$$R_{t}=\frac{I_{t}}{\sum_{s=1}^{t}I_{t-s}w_{s}},$$

where $I_{t}$ is the number incident infections on day t and $w_{s}$ is the generation interval at time since infection s. An individual’s relative infectiousness depends on generation interval and time since infection (s) [45]. Here the term $\sum_{s=1}^{t}I_{t-s}w_{s}$ describes the sum of infection incidence up to time step (*t* − 1) weighted by the current infectiousness ($w_{s}$) of individuals who became infected s days in the past, and who may be shedding the virus now. The standard assumption is that $w_{s}$ follows a discretized gamma distribution [45]. However, since the infection time may not be accurately observed, the measurement of generation time becomes difficult [46] and so the time of symptom onset is usually used to estimate the distribution of serial interval (SI), which is the time interval between dates of symptom onset among two successive cases in a disease transmission chain [45]. We assumed the generation interval as equal to SI and that SI follows a gamma distribution with a mean of 5.2 days and a standard deviation of 1.72 days [41]. We then obtained the $R_{t}$ estimates over weekly time interval within a Bayesian framework using EpiEstim R package in R language [45]. We reported the mean and 95% credible interval (CrL).

## 3. Results

**Figure 1:**
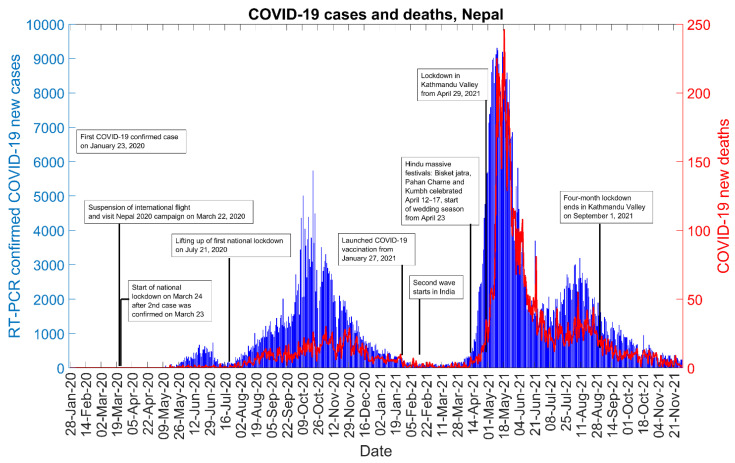
Epidemic curve of the COVID-19 pandemic in Nepal as of 30 November 2021.

As of 30 November 2021, Nepal reported a total of 821,366 RT-PCR positive COVID-19 cases and a total of 11,526 deaths. Figure 1 shows the incidence curve of COVID-19 cases and deaths in Nepal as of 30 November 2021. The COVID-19 epidemic curve shows three distinct phases: an initial growth phase occurring during late-May to late-June 2020, the first wave occurring from early-August 2020 to mid-January 2021, and the second wave taking off in mid-April 2021 (Figure 1). The first wave started to peak after the lifting of the first national lockdown, which lasted about four months. The second wave was preceded by the second wave of COVID-19 in India, a celebration of Hindu massive festivals, and the start of wedding season in Nepal. The second wave came to a decline in late June 2021, but it resurged a few weeks later (Figure 1).

### 3.1. Model Calibration and Forecasting Performance

**Figure 2:**
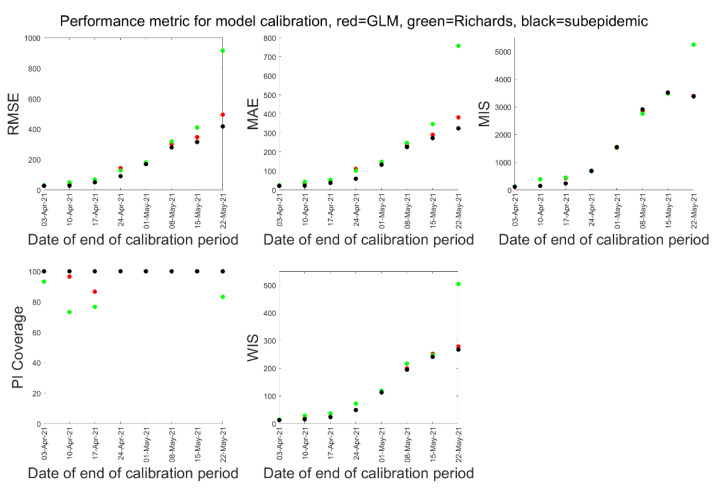
Comparison of performance metric during model calibration. Red dots indicate performance of the GLM model, green dots indicate performance of the Richards model, and the black dots indicate performance of the sub-epidemic model.

Figure 2 shows a comparison of the results for five performance metrics of model calibration. We found that during the model calibration, sub-epidemic model outperformed the GLM and Richards model based on RMSE, MAE, PI-Coverage, and WIS in all the 8-model calibrations conducted.

In Table 2, we present the comparison of performance metrics of the three models for 10-days ahead forecasts. For 10-days ahead forecast, GLM model performed better in RMSE and MIS, while sub-epidemic model performed better in PI-Coverage. Overall, we saw that in the first three forecast periods, the sub-epidemic model out-performed the GLM and Richards models in all the metrics. Likewise, the Richards model out-performed GLM and the sub-epidemic model in all the metrics in the fifth and sixth forecast period, while the GLM model performed better in the eighth forecast period (Table 2).

Table 3 presents the comparison of performance metrics of the three models for 20-days ahead forecast. For 20-days ahead forecast, GLM model performed better in RMSE, and sub-epidemic model performed better in MIS, and PI-coverage in the majority of the forecast periods. Similar to the 10-days ahead forecast, the 20-days ahead forecast also observed the Richards model performing better in most of the metrics.

**Figure 3:**
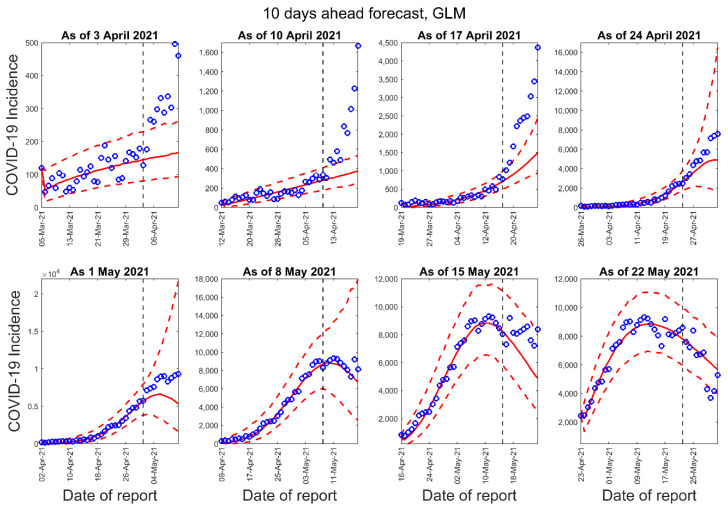
Ten-days ahead forecasts of the COVID-19 epidemic curves in Nepal by calibrating the GLM model for eight different periods. Blue circles correspond to the data points; the solid red line indicates the best model fit, and the red dashed lines represent the 95% prediction interval. The vertical black dashed line represents the time of the start of the forecast period.

**Figure 4:**
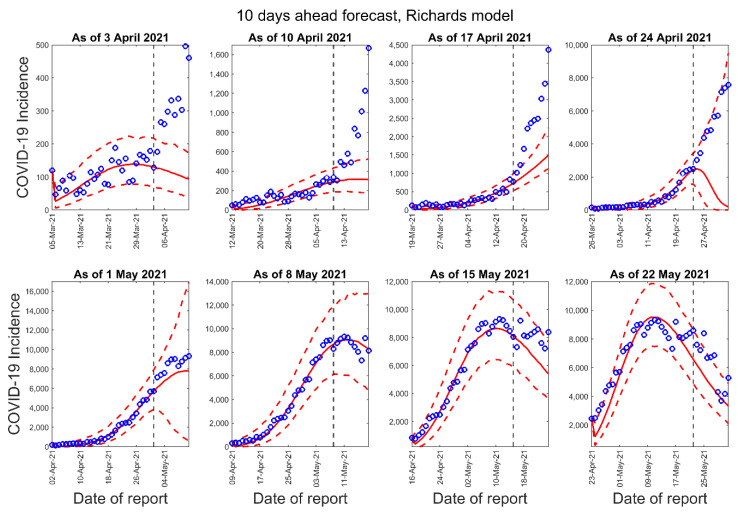
Ten-days ahead forecasts of the COVID-19 epidemic curves in Nepal by calibrating the Richards model for eight different periods. Blue circles correspond to the data points; the solid red line indicates the best model fit, and the red dashed lines represent the 95% prediction interval. The vertical black dashed line represents the time of the start of the forecast period.

**Figure 5:**
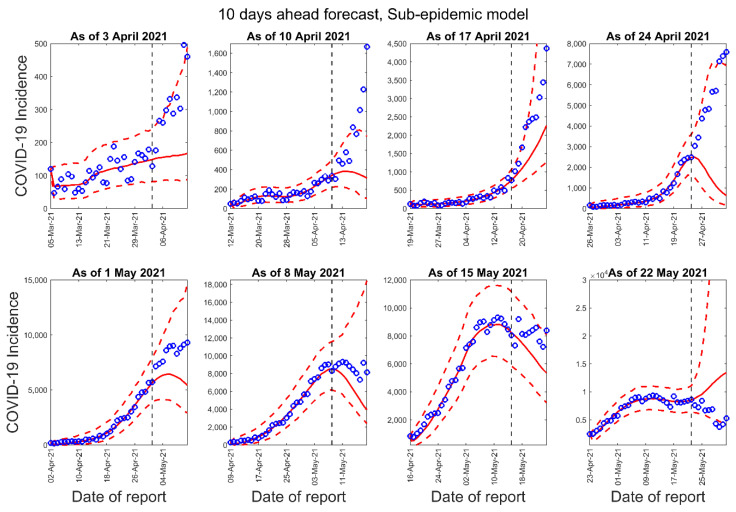
Ten-days ahead forecasts of the COVID-19 epidemic curves in Nepal by calibrating the sub-epidemic model for 8 different periods. Blue circles correspond to the data points; the solid red line indicates the best model fit, and the red dashed lines represent the 95% prediction interval. The vertical black dashed line represents the time of the start of the forecast period.

**Figure 6:**
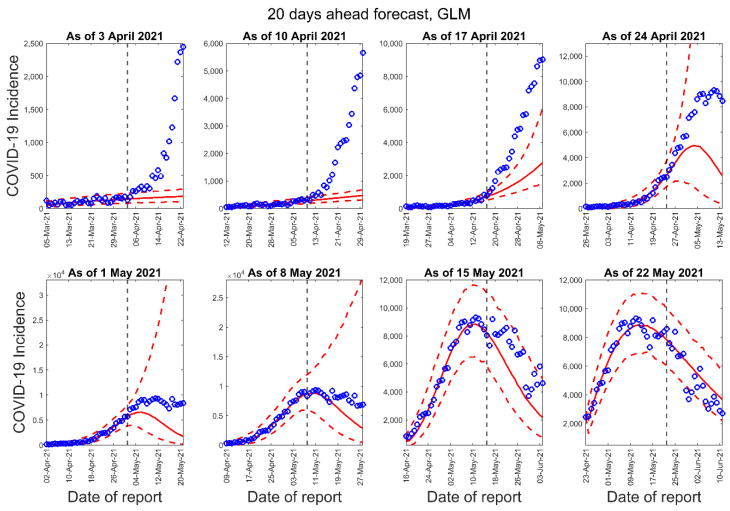
Twenty-days ahead forecasts of the COVID-19 epidemic curves in Nepal by calibrating the GLM model for eight different periods. Blue circles correspond to the data points; the solid red line indicates the best model fit, and the red dashed lines represent the 95% prediction interval. The vertical black dashed line represents the time of the start of the forecast period.

**Figure 7:**
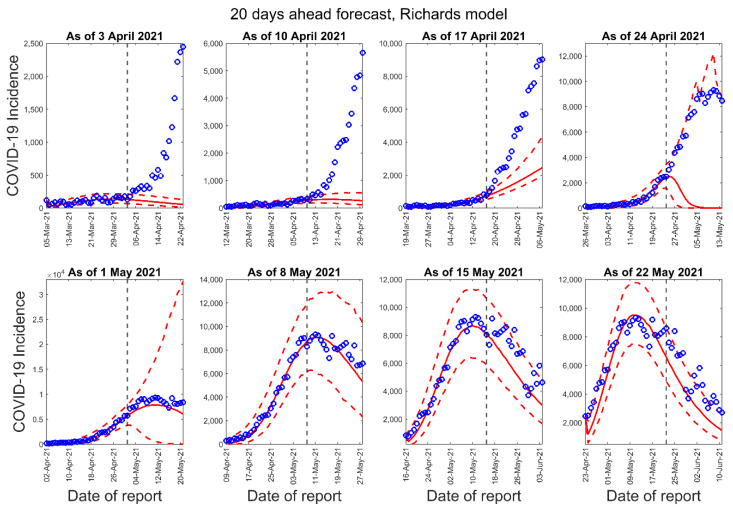
Twenty-days ahead forecasts of the COVID-19 epidemic curves in Nepal by calibrating the Richards model for eight different periods. Blue circles correspond to the data points; the solid red line indicates the best model fit, and the red dashed lines represent the 95% prediction interval. The vertical black dashed line represents the time of the start of the forecast period.

**Figure 8:**
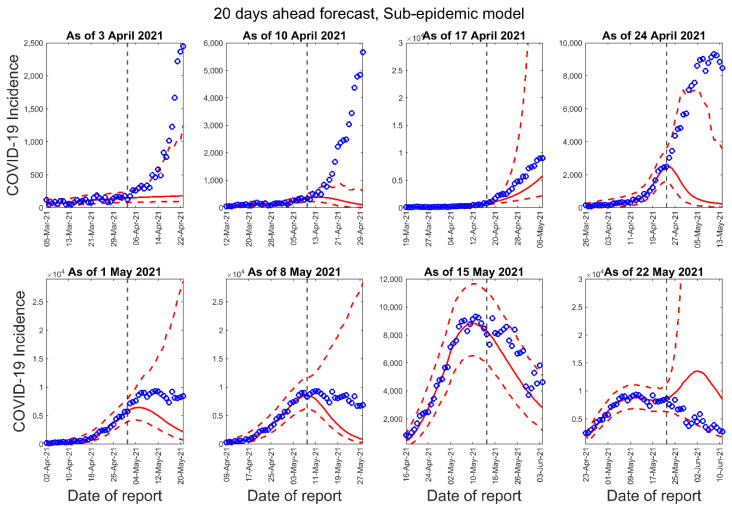
Twenty-days ahead forecasts of the COVID-19 epidemic curves in Nepal by calibrating the sub-epidemic model for eight different periods. Blue circles correspond to the data points; the solid red line indicates the best model fit, and the red dashed lines represent the 95% prediction interval. The vertical black dashed line represents the time of the start of the forecast period.

Figure 3, Figure 4 and Figure 5 below show the 10-days ahead forecast for 8 different sequential periods for three dynamic phenomenological models. Similarly, Figure 6, Figure 7 and Figure 8 show the 20-days ahead forecast for the same forecast periods for the three models.

### 3.2. Estimate of Reproduction Number, $R_{t}$ from Case Incidence Data Using GGM

The reproduction number for the early ascending growth phase of the initial mild wave of epidemic from the case incidence data (25 May to 23 June 2020) using GGM was estimated at $R_{t}$~1.1 (95% CI: 1.1, 1.2) for the national data. The growth rate was 13 (95% CI: 7, 20) and the deceleration of growth rate parameter (p) was estimated at 0.41 (95% CI: 0.35, 0.48). Likewise, for the first 30 days of the first wave from 1 August to 30 August 2020, the reproduction number was estimated at 1.1 (95% CI: 1.1, 1.2), the growth rate was estimated at 15 (95% CI: 8.5, 20), and the deceleration of growth rate parameter was estimated at 0.43 (95% CI: 0.39, 0.49). The deceleration of growth rate parameter for the initial mild wave and the first wave (p~0.41–0.43) indicate almost a linear pattern of epidemic trajectory of COVID-19 in Nepal.

**Figure 9:**
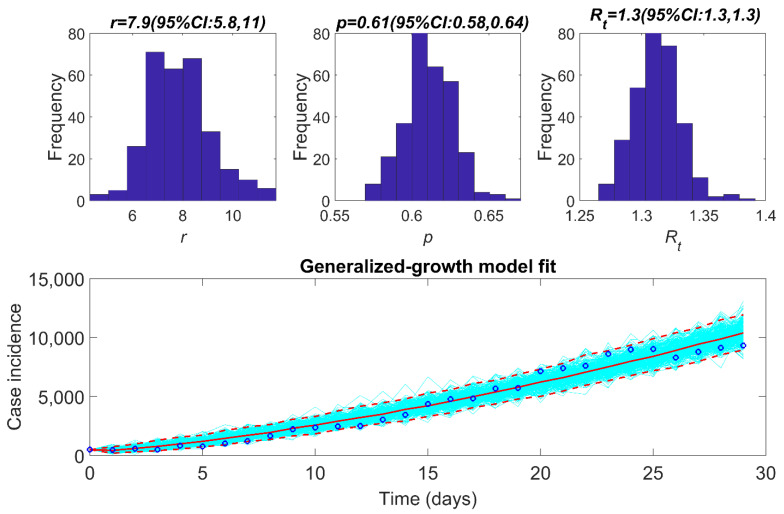
Upper panel: reproduction number for Nepal for the early phase of the second wave of epidemic (12 April 2021–11 May 2021) with 95% CI estimated using the GGM model. The estimated reproduction number of the COVID-19 epidemic in Nepal as of 12 May 2020, is 1.3 (95% CI: 1.3, 1.3). The growth rate parameter, r, is estimated at 7.9 (95% CI: 5.8, 11) and the deceleration of growth parameter, p, is estimated at 0.61 (95% CI:0.58, 0.64). Lower panel: the lower panel shows the GGM fit to the case incidence data for the first 30 days of the second wave from 12 April 2021–11 May 2021.

**Figure 10:**
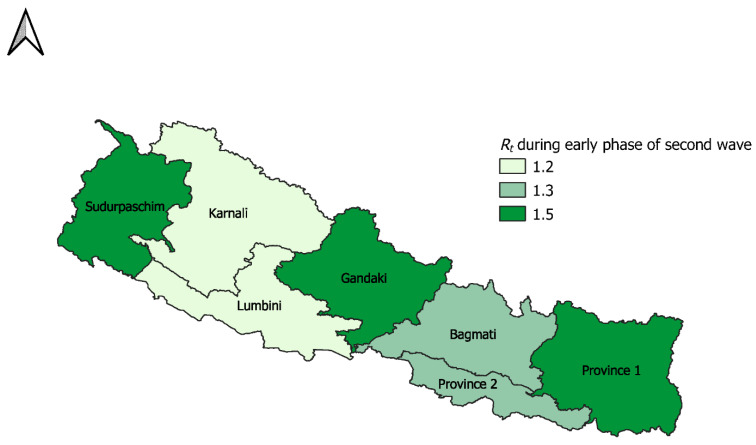
Map showing reproduction number at province level, for first 30 days of the second wave (12 April–11 May 2021).

For the second major wave, we estimated the reproduction number ($R_{t}$) at national and provincial level. For the early phase of the second wave (first 30 days), $R_{t}$ at national level was 1.3 (95% CI: 1.3, 1.4) with the growth rate of 7.9 (95% CI: 5.8, 11) and p of 0.61 (95% CI: 0.58, 0.64) (Figure 9, Table 4), indicating a sub-exponential growth dynamic of COVID-19 pandemic. At provincial level, while province 1, Gandaki, and Sudurpaschim had the highest $R_{t}$ at 1.5, Lumbini and Karnali regions observed the lowest $R_{t}$ at 1.2 (Figure 10, Table 4). For province 1, Bagmati, Gandaki, and Sudurpaschim, the deceleration of growth rate parameter indicated sub-exponential growth dynamics with estimated between p~0.61–0.72. For other provinces, p~0.51–0.58 indicated an almost linear trajectory of the COVID-19 epidemic (Table 4).

We also estimated the reproduction number for the 30 days period from 10 July–8 August 2021, for the early ascending phase of the resurgence that started to peak from early July 2021. The reproduction number for the early ascending growth phase of the resurgence phase after the second wave from the case incidence data (10 July to 8 August 2021) using GGM was estimated at $R_{t}$~1.2 (95% CI: 1.2, 1.2) for the national level. The growth rate was 18 (95% CI: 12, 20) and the deceleration of growth rate parameter (*p*) was estimated at 0.47 (95% CI: 0.46, 0.52).

### 3.3. Estimate of Instantaneous Reproduction Number, $R_{t}$

**Figure 11:**
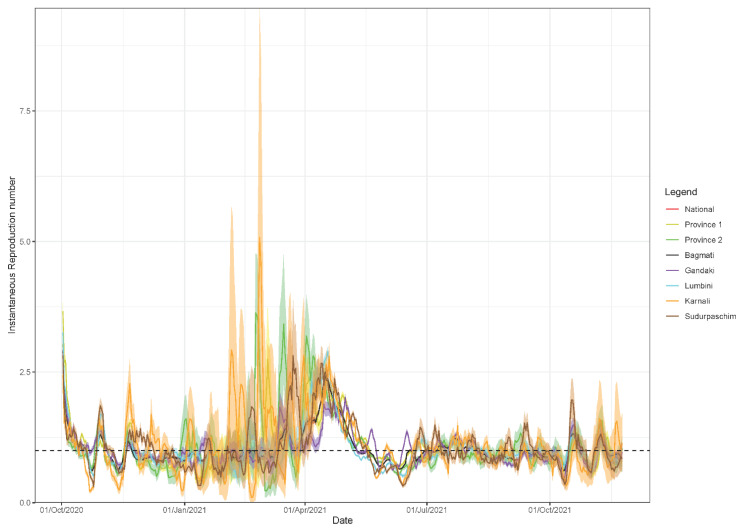
Instantaneous reproduction number for the period from 1 October 2020–30 November 2021.

Figure 11 shows the weekly instantaneous reproduction number at national and provincial level for the period from 1 October 2020 to 30 September 2021. The reproduction number was estimated at, $R_{t}$~2.5 (95% CrI: 2.4, 2.5) on 16 April 2021, which declined to 0.99 (95% CI: 0.98, 1.0) on 10 May 2021, and increased to 1.04 (95% CI:1.02, 1.06) on 9 July 2021 at national level (Figure 11). At the provincial level, highest $R_{t}$ was observed in Karnali region with an estimate of $R_{t}$~5.08 (95% CrI: 2.04, 9.48) on 26 February 2021. The instantaneous reproduction number fluctuated at around ~1 from December 2020 to January 2021 for all the regions. Similarly, instantaneous reproduction has remained consistently below 1 for national level and fluctuated between 0–1.5 at provincial level since 8 August 2021.

### 3.4. Analysis of Mobility Data

**Figure 12:**
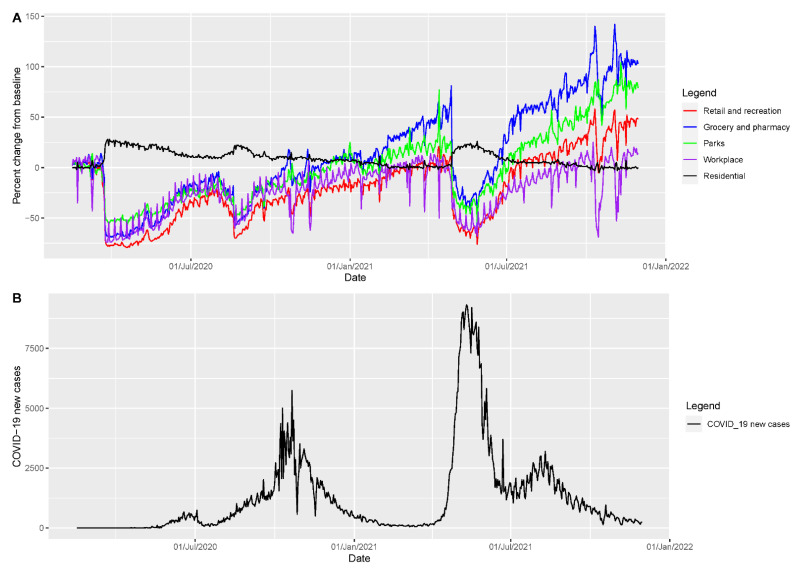
(**A**) Google mobility trends data showing the variation in movement of people across the following five categories: grocery and pharmacy (blue curve), parks (green curve), residential (black curve), retail and creation (red curve), and workplace (purple curve) among the population in Nepal. (**B**) COVID-19 incidence curve in Nepal by the dates of report as of 30 November 2021.

The curves of mobility from Google data tracked in the form of visits to retail and recreation, grocery and pharmacy, parks, and workplaces all follow the same pattern, showing inclining trends from late May to late June 2020, which correspond to the peak of initial rise in cases of COVID-19. The mobility curve started to incline again in August, which corresponded to the early phase of the first wave. The second wave started soon after there was a peak in mobility around parks and grocery and pharmacy. During the peak in the second wave, mobility declined rapidly in all the areas except residential, which corresponds to the lockdown by government as an intervention to contain the epidemic (Figure 12).

## 4. Discussion

Reliable forecasting tools are of critical importance in guiding public health policy during the period of an ongoing pandemic [47]. Appropriate short-term forecasts not only help assess the impact of interventions but also help guide the distribution of limited resources. In this study we compared the performance of three different phenomenological models for short term forecast of COVID-19 cases based on the estimates derived using case incidence data at national level. We performed eight different week-to-week sequential forecasts of 10-days and 20-days. Our findings indicate the better performance of sub-epidemic model based on five performance metrics during the model calibration phase. However, for the 10-days and 20-days ahead forecast performance, we found the sub-epidemic model performing better in the initial forecast periods and the Richards model performing better in the later forecast periods. Our finding is in line with previous studies indicating that the sub-epidemic model frequently outperforms the GLM and Richards model [48]. Our forecast estimates from the three phenomenological models indicate a declining trend of COVID-19 cases in Nepal as of June 2021.

The early estimate of $R_{t}$ in different epidemic waves reflect a sustained transmission of COVID-19 in Nepal with $R_{t}$ above 1. We report almost a linear pattern of COVID-19 incidence during the initial transmission phase and the first waves in Nepal (deceleration of growth parameter *p*~0.41–0.43) with a reproduction number at 1.1 (95% CI: 1.1, 1.2). We also report a sub-exponential growth pattern in the second wave with a deceleration of growth parameter *p* at 0.61 (95% CI: 0.58, 0.64) and a corresponding reproduction number at 1.3 (95% CI: 1.3, 1.3). We found much variation in the reproduction number, growth rate, and deceleration of growth rate parameters at the provincial level, with province 1, Gandaki and Sudurpaschim provinces having $R_{t}$ at 1.5 during the initial phase of the major second wave. Our estimate of reproduction number is less than that of India [49,50] and comparable with early estimates of reproduction number reported from Mexico [48] and South Korea [51], and higher than that of the early estimates reported from Singapore [52].

As of 24 August 2021, the SARS Cov-2 variants of concern (VOC) reported from Nepal include Alpha and Delta variants [53]. The Delta variant is at least twice as contagious as previous variants [54]. The national public health laboratory of Nepal confirmed the detection of the Delta variant in 47 of the 48 swab samples collected from infected individuals from different parts of the county from 9 May to 3 June 2021, and in nine of these samples, the highly infectious new sub-lineage K417N (also called AY.1) was detected [55], indicating the presence of Delta variant since at least May 2021. However, unlike expected higher value of reproduction number for the period with the circulating Delta variant, our estimate of instantaneous reproduction number did not show an increase in $R_{t}$ above 1, both at national level and provincial level since 25 May–12 June 2021 (Figure 11). This could be due to the vaccination efforts by the country during early/mid 2021 [56,57] and also due to inadequate COVID-19 testing rates around that time, due to a large number of cases that may have been undetected [9].

**Figure 13:**
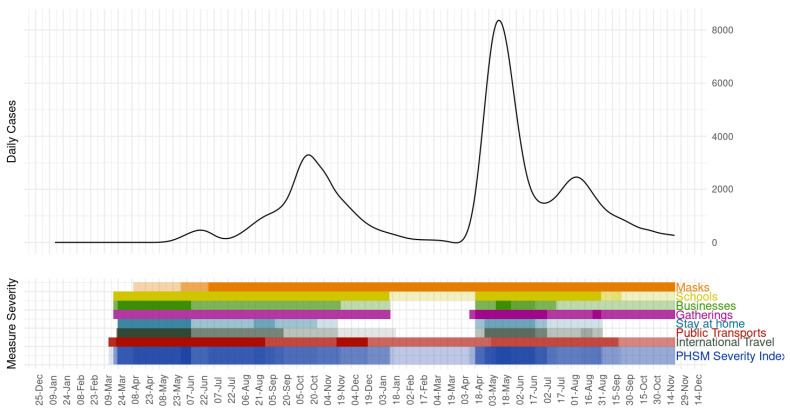
Daily COVID-19 cases over severity of public health and social measures (PHSM) in Nepal (March 2021–23 November 2021). Figure source: PHSM dashboard published by WHO regional office for South East Asia [58]. Indicators and PHSM severity index are assigned a color. The shading of that color is based on the severity score. The darkest shade represents the most severe measure/s (value of 100) and a lack of color represents no measure (value of 0).

Our analysis of mobility trend shows the peak in mobility across different areas relative to the trajectory of the COVID-19 incidence (Figure 12). The increase in mobility before the third wave corresponds to the start of wedding season [14], celebration of Hindu massive festivals in Nepal, crowded movement of people in the country, a lack of adherence to social distancing guidelines [13], and the start of second wave in India (Figure 1). Our findings from google mobility data are in line with the country’s severity index of public health and social measures (PHSM) published by the WHO regional office for South East Asia (Figure 13) [58]. Figure 13 shows that decline in the severity index for PHSM was followed by the surge in daily COVID-19 cases. The lifting of bans on businesses, gatherings, stay at home, and public transport during February and March 2021 preceded the start of second wave during April 2021 in Nepal (Figure 1, Figure 13).

Nepal depends on India for most of its supplies, including medical equipment, liquid oxygen, and vaccines. Therefore, when the situation deteriorated in India’s health system with a total of 26,031,991 COVID-19 cases and 291,331 deaths, as of 21 May 2021, Nepal struggled to find alternate sources of supplies beyond India [15]. India’s surge in COVID-19 cases impacted the availability of COVID-19 vaccines in Nepal. Nepal launched its COVID-19 vaccination program on 27 January 2021 after receiving a first batch of one million doses of AstraZeneca (Covishield) vaccines produced by the Serum Institute of India [15]. Nepal also received doses from Covax and from China [15]. However, Nepal’s attempt to buy additional doses of AstraZeneca vaccine from India failed due to the increase in demand in that country [16]. As of 30 November 2021, 27.8% of the total population in Nepal have been fully vaccinated and 32.5% have received at least one dose [7]. Given the vaccination rate, fragile health system, direct impact of the COVID-19 trajectory in India (in terms of cases flow due to open border as well as in terms of management as seen during the vaccination), and a sustained COVID-19 transmission, there is a need to implement non-pharmaceutical measures in order to reduce the reproduction number.

Our study has several public health implications. Short-term forecasts using the sub-epidemic and Richards models yield the most reliable short-term forecasts. This can be useful in planning the interventions during the early growth phase of the epidemic at present and for similar epidemics in the future. Furthermore, the analysis of the sub-national estimates of the reproduction numbers could help prioritize subpopulations on interventions, especially for a resource-scarce country such as Nepal.

Our study has some limitations. The case positivity rate fluctuated around 10% during the second week of April 2021 and rose to 73% between mid-April and mid-May [16], suggesting that only severe symptomatic cases were being tested and a much larger population could have been infected. Therefore, our estimates of reproduction number may be underestimated. Similarly, we used only RT-PCR positive case counts in our study while the national situation reports provide the total number of cases includes both RT-PCR and antigen positive cases. Of note, the descriptive analysis of the data in most of situational reports included only the RT-PCR positive cases. Considering only RT-PCR cases will make our findings comparable with other countries [59]. Moreover, the cases incidence and mortality data used in this study are based on the reporting date, rather than date of symptom onset or date of death. Therefore, the difference in the report date and actual date of event occurrence, including the differences in testing rates and reporting delay, might affect our model estimates and estimates of reproduction number.

## 5. Conclusions

The reproduction number in Nepal has been fluctuating around 1 since January 2021 indicating a sustained virus transmission in the country. We report a sub-exponential growth dynamic during the early phase of the second wave with a reproduction number at 1.3 at the national level with province 1, Gandaki and Sudurpaschim provinces having higher reproduction numbers ($R_{t}$ at 1.5) during the initial phase of the major second wave. The increase in mobility in the country appears to be positively correlated with COVID-19 transmission. The sub-epidemic and Richards model provide reasonable short-term projections of the COVID-19 trajectory in Nepal and indicate a declining trend of COVID-19 cases in the country until June 2021. Simple mathematical models can provide reliable short-term projections, which are crucial for public health planning.

## Figures and Tables

**Table 1:** Calibration period and forecast period for each forecast.

Forecast Number	Calibration Period for the GLM, Richards, and Sub-Epidemic Model	Number of Days in the Calibration Period	Forecast Period for 10-Days Ahead Forecast	Forecast Period for 20-Days Ahead Forecast
1	5 March 2021–3 April 2021	30	4 April 2021–13 April 2021	4 April 2021–23 April 2021
2	12 March 2021–10 April 2021	30	11 April 2021–20 April 2021	11 April 2021–30 April 2021
3	19 March 2021–17 April 2021	30	18 April 2021–27 April 2021	18 April 2021–7 May 2021
4	26 March 2021–24 April 2021	30	25 April 2021–4 May 2021	25 April 2021–14 May 2021
5	2 April 2021–1 May 2021	30	2 May 2021–11 May 2021	2 May 2021–21 May 2021
6	9 April 2021–8 May 2021	30	9 May 2021–18 May 2021	9 May 2021–28 May 2021
7	16 April 2021–15 May 2021	30	16 May 2021–25 May 2021	16 May 2021–4 June 2021
8	23 April 2021–22 May 2021	30	23 May 2021–June 1 2021	23 May 2021–11 June 2021

**Table 2:** Comparison of performance metric during 10-days ahead forecast.

	3 April 2021	10 April 2021	17 April 2021	24 April 2021	1 May 2021	8 May 2021	15 May 2021	22 May 2021
	RMSE							
GLM	183.77	580.17	1.51 × 10^3^	1.57 × 10^3^	2.41 × 10^3^	805.31	1.93 × 10^3^	1.23 × 10^3^
Richards	228.92	615.59	1.51 × 10^3^	4.68 × 10^3^	1.13 × 10^3^	580.1	1.61 × 10^3^	1.68 × 10^3^
Sub-epidemic	184.21	591.12	1.07 × 10^3^	4.44 × 10^3^	2.41 × 10^3^	2.64 × 10^3^	1.68 × 10^3^	6.03 × 10^3^
	**MAE**							
GLM	163.17	446.36	1.32 × 10^3^	1.28 × 10^3^	2.23 × 10^3^	549.5	1.74 × 10^3^	891.01
Richards	206.49	472.57	1.31 × 10^3^	4.02 × 10^3^	1.09 × 10^3^	425.69	1.47 × 10^3^	1.44 × 10^3^
Sub-epidemic	163.14	431.97	934.05	3.88 × 10^3^	2.26 × 10^3^	2.30 × 10^3^	1.53 × 10^3^	5.18 × 10^3^
	**MIS**							
GLM	3.34 × 10^3^	1.30 × 10^4^	3.18 × 10^4^	7.35 × 10^2^	1.12 × 10^4^	1.06 × 10^4^	6.14 × 10^3^	7.67 × 10^3^
Richards	5.37 × 10^3^	1.26 × 10^4^	3.44 × 10^4^	5.80 × 10^3^	1.02 × 10^4^	7.12 × 10^3^	7.37 × 10^3^	1.18 × 10^4^
Sub-epidemic	5.97 × 10^3^	7.06 × 10^3^	3.40 × 10^3^	9.49 × 10^3^	7.76 × 10^3^	1.01 × 10^4^	6.25 × 10^3^	4.96 × 10^4^
	**PI-Coverage**							
GLM	10	10	10	100	100	100	90	80
Richards	10	30	10	100	100	100	90	60
Sub-epidemic	80	60	90	80	100	100	90	70
	**WIS**							
GLM	132.41	401.63	1.12 × 10^3^	625.49	1.22 × 10^3^	600.13	1.05 × 10^3^	625.39
Richards	179.84	412.37	1.13 × 10^3^	2.88 × 10^3^	723.83	440.77	919.38	9.74 × 10^2^
Sub-epidemic	114.41	341.45	538.37	2.74 × 10^3^	1.33 × 10^3^	1.37 × 10^3^	8.97 × 10^2^	3.30 × 10^3^

*Dates in each column indicate date of the end of 30-days calibration period*.

**Table 3:** Comparison of performance metric during 20-days ahead forecast.

	3 April 2021	10 April 2021	17 April 2021	24 April 2021	1 May 2021	8 May 2021	15 May 2021	22 May 2021
	RMSE							
GLM	985.56	2.39 × 10^3^	3.65 × 10^3^	3.80 × 10^3^	4.03 × 10^3^	2.37 × 10^3^	2.08 × 10^3^	1.11 × 10^3^
Richards	1.06 × 10^3^	2.50 × 10^3^	3.81 × 10^3^	7.07 × 10^3^	1.18 × 10^3^	937.85	1.57 × 10^3^	1.68 × 10^3^
Sub-epidemic	988.99	2.57 × 10^3^	2.28 × 10^3^	6.80 × 10^3^	3.97 × 10^3^	4.54 × 10^3^	1.69 × 10^3^	7.00 × 10^3^
	**MAE**							
GLM	673.39	1.79 × 10^3^	3.06 × 10^3^	3.18 × 10^3^	3.65 × 10^3^	1.85 × 10^3^	1.88 × 10^3^	898.59
Richards	745.56	1.87 × 10^3^	3.16 × 10^3^	6.42 × 10^3^	1.08 × 10^3^	739.33	1.39 × 10^3^	1.50 × 10^3^
Sub-epidemic	675.72	1.90 × 10^3^	1.96 × 10^3^	6.20 × 10^3^	3.65 × 10^3^	4.06 × 10^3^	1.49 × 10^3^	6.47 × 10^3^
	**MIS**							
GLM	2.34 × 10^3^	6.56 × 10^4^	7.29 × 10^4^	2.60 × 10^4^	2.41 × 10^4^	1.65 × 10^4^	6.27 × 10^3^	5.89 × 10^3^
Richards	2.69 × 10^4^	6.67 × 10^4^	9.18 × 10^4^	9.11 × 10^3^	1.78 × 10^4^	7.67 × 10^3^	7.37 × 10^3^	1.47 × 10^4^
Sub-epidemic	1.07 × 10^3^	6.07 × 10^4^	2.44 × 10^4^	7.86 × 10^4^	1.38 × 10^4^	1.64 × 10^4^	6.02 × 10^3^	3.81 × 10^5^
	**PI-Coverage**							
GLM	5	5	5	100	100	100	95	85
Richards	5	10	5	95	100	100	85	45
Sub-epidemic	45	30	95	35	100	100	90	85
	**WIS**							
GLM	639.32	1.73 × 10^3^	2.60 × 10^3^	1.64 × 10^3^	2.19 × 10^3^	1.17 × 10^3^	1.14 × 10^3^	575.45
Richards	719.45	1.79 × 10^3^	2.78 × 10^3^	4.93 × 10^3^	895.16	534.59	899.52	1.05 × 10^3^
Sub-epidemic	556.18	1.75 × 10^3^	1.16 × 10^3^	4.98 × 10^3^	2.32 × 10^3^	2.77 × 10^3^	9.12 × 10^2^	7.12 × 10^3^

*Dates in each column indicate date of the end of 30-days calibration period*.

**Table 4:** Comparison of reproduction number estimated using GGM for provinces, for first 30 days of second wave (12 April–11 May 2021).

Region	Reproduction Number (95% CI)	Growth Rate (95% CI)	Deceleration of Growth Parameter (95% CI)
National	1.3 (1.3, 1.3)	7.9 (5.8, 11)	0.61 (0.58, 0.64)
Province 1	1.5 (1.4, 1.6)	1.2 (0.77, 1.8)	0.72 (0.67, 0.79)
Province 2	1.3 (1.2, 1.3)	3.1 (2.1, 4.4)	0.58 (0.53, 0.63)
Bagmati	1.3 (1.3, 1.4)	6.6 (4.0, 9.7)	0.61 (0.56, 0.66)
Gandaki	1.5 (1.3, 1.8)	1.2 (0.53, 2.2)	0.73 (0.63, 0.84)
Lumbini	1.2 (1.2, 1.3)	8.7 (4.1, 16)	0.53 (0.46, 0.61)
Karnali	1.2 (1.1, 1,4)	6.1 (1.4, 15)	0.51 (0.36, 0.68)
Sudurpaschim	1.5 (1.3, 1.7)	1.3 (0.59, 2.4)	0.71 (0.61, 0.82)

## Data Availability

The data used in this study are publicly available from COVID-19 situation reports published by Ministry of Health and Population of the government of Nepal.

## References

[1] (2021). Demographia World Urban Areas. http://www.demographia.com/db-worldua.pdf.

[2] Kugelman M. (2021). How COVID-19 Has Shaped South Asia. Foreign Policy.

[3] Pecho-Silva S., Barboza J.J., Navarro-Solsol A.C., Rodriguez-Morales A.J., Bonilla-Aldana D.K., Panduro-Correa V. (2021). SARS-CoV-2 Mutations and Variants: What do we know so far?. Microbes Infect. Chemother..

[4] WHO (2021). COVID-19 Weekly Epidemiological Update-37.

[5] COVID Variants: What You Should Know. https://www.hopkinsmedicine.org/health/conditions-and-diseases/coronavirus/a-new-strain-of-coronavirus-what-you-should-know.

[6] WHO (2021). COVID-19 Dashboard.

[7] MoHP Nepal (2021). COVID-19 Situation Update 30 November 2021.

[8] Dhakal S., Karki S. (2020). Early epidemiological features of COVID-19 in Nepal and public health response. Front. Med..

[9] Hollingsworth J., Jeong S., Thapa A. (2021). Nepal’s cases skyrocket, prompting concern the country’s outbreak could mimic India’s. https://edition.cnn.com/2021/05/06/asia/nepal-covid-outbreak-intl-hnk-dst/index.html.

[10] Dalal S. (2020). Relations between India and Nepal in Covid-19 situation. J. Hist. Archaeol. Anthropol. Sci..

[11] BW Business World Nepal Re-Opens Border with India with Restrictions. http://www.businessworld.in/article/Nepal-re-opens-border-with-India-with-restrictions/29-01-2021-371457/.

[12] Alam J. Virus Surge, Vaccine Shortages Spread beyond India’s Borders. https://apnews.com/article/india-europe-business-global-trade-coronavirus-e95f0515b68ed20ea1f0a53bdea3ffae.

[13] The Guardian ‘It’s as If There’s No Covid’: Nepal Defies Pandemic Amid a Broken Economy. https://www.theguardian.com/global-development/2021/feb/11/its-as-if-theres-no-covid-nepal-defies-pandemic-amid-a-broken-economy.

[14] Majority of Those Infected in Chitwan Attended Wedding, Bratabandha and Feasts. https://www.kantipurhealth.com/archives/5055.

[15] Ethirajan A. As India Halts Vaccine Exports, Nepal Faces Its Own Covid Crisis. https://www.bbc.com/news/world-asia-57055209.

[16] Adhikari D. Nepal Struggles with a Surge in COVID-19 Cases. https://www.devex.com/news/nepal-struggles-with-a-surge-in-covid-19-cases-99953.

[17] Nepal: Act to Avert Looming Covid-19 Disaster. https://www.hrw.org/node/378679/printable/print.

[18] Jewell N.P., Lewnard J.A., Jewell B.L. (2020). Predictive mathematical models of the COVID-19 pandemic: Underlying principles and value of projections. JAMA.

[19] Chowell G., Hincapie-Palacio D., Ospina J., Pell B., Tariq A., Dahal S., Moghadas S., Smirnova A., Simonsen L., Viboud C. (2016). Using phenomenological models to characterize transmissibility and forecast patterns and final burden of Zika epidemics. PLoS Curr..

[20] Pell B., Kuang Y., Viboud C., Chowell G. (2018). Using phenomenological models for forecasting the 2015 Ebola challenge. Epidemics.

[21] Election Commission Provincial Map of Nepal. https://www.election.gov.np/uploads/Pages/1564381682_np.pdf.

[22] Aljazeera Hundreds of Nepalese Stuck at India Border Amid COVID-19 Lockdown. https://www.aljazeera.com/news/2020/4/1/hundreds-of-nepalese-stuck-at-india-border-amid-covid-19-lockdown.

[23] Government of Nepal Covid-19 Situation Reports.

[24] Google Covid-19 Community Mobility Report for Nepal. https://www.google.com/covid19/mobility/.

[25] Shanafelt D.W., Jones G., Lima M., Perrings C., Chowell G. (2018). Forecasting the 2001 foot-and-mouth disease epidemic in the UK. EcoHealth.

[26] Roosa K., Lee Y., Luo R., Kirpich A., Rothenberg R., Hyman J.M., Yan P., Chowell G. (2020). Short-term forecasts of the COVID-19 epidemic in Guangdong and Zhejiang, China: 13–23 February 2020. J. Clin. Med..

[27] Roosa K., Lee Y., Luo R., Kirpich A., Rothenberg R., Hyman J., Yan P., Chowell G. (2020). Real-time forecasts of the COVID-19 epidemic in China from 5 to 24 February 2020. Infect. Dis. Model..

[28] Richards F. (1959). A flexible growth function for empirical use. J. Exp. Bot..

[29] Chowell G., Tariq A., Hyman J.M. (2019). A novel sub-epidemic modeling framework for short-term forecasting epidemic waves. BMC Med..

[30] Chowell G. (2017). Fitting dynamic models to epidemic outbreaks with quantified uncertainty: A primer for parameter uncertainty, identifiability, and forecasts. Infect. Dis. Model..

[31] Wang X.-S., Wu J., Yang Y. (2012). Richards model revisited: Validation by and application to infection dynamics. J. Theor. Biol..

[32] Banks H.T., Hu S., Thompson W.C. (2014). Modeling and Inverse Problems in the Presence of Uncertainty.

[33] Roosa K. (2020). Comparative Assessment of Epidemiological Models for Analyzing and Forecasting Infectious Disease Outbreaks. Ph.D. Thesis.

[34] Roosa K., Chowell G. (2019). Assessing parameter identifiability in compartmental dynamic models using a computational approach: Application to infectious disease transmission models. Theor. Biol. Med. Model..

[35] Gneiting T., Raftery A.E. (2007). Strictly proper scoring rules, prediction, and estimation. J. Am. Stat. Assoc..

[36] Kuhn M.J.K. (2013). Applied Predictive Modeling.

[37] Cramer E.Y., Lopez V.K., Niemi J., George G.E., Cegan J.C., Dettwiller I.D., England W.P., Farthing M.W., Hunter R.H., Lafferty B. (2021). Evaluation of individual and ensemble probabilistic forecasts of COVID-19 mortality in the US. medRxiv.

[38] Bracher J., Ray E.L., Gneiting T., Reich N.G. (2021). Evaluating epidemic forecasts in an interval format. PLoS Comput. Biol..

[39] Inglesby T.V. (2020). Public health measures and the reproduction number of SARS-CoV-2. JAMA.

[40] Pan A., Liu L., Wang C., Guo H., Hao X., Wang Q., Huang J., He N., Yu H., Lin X. (2020). Association of public health interventions with the epidemiology of the COVID-19 outbreak in Wuhan, China. JAMA.

[41] Ganyani T., Kremer C., Chen D., Torneri A., Faes C., Wallinga J., Hens N. (2020). Estimating the generation interval for coronavirus disease (COVID-19) based on symptom onset data, March 2020. Eurosurveillance.

[42] Nishiura H., Chowell G. (2014). Early transmission dynamics of Ebola virus disease (EVD), West Africa, March to August 2014. Eurosurveillance.

[43] Nishiura H., Chowell G. (2009). The effective reproduction number as a prelude to statistical estimation of time-dependent epidemic trends. Mathematical and Statistical Estimation Approaches in Epidemiology.

[44] Paine S., Mercer G., Kelly P., Bandaranayake D., Baker M., Huang Q., Mackereth G., Bissielo A., Glass K., Hope V. (2010). Transmissibility of 2009 pandemic influenza A (H1N1) in New Zealand: Effective reproduction number and influence of age, ethnicity and importations. Eurosurveillance.

[45] Cori A., Ferguson N.M., Fraser C., Cauchemez S. (2013). A new framework and software to estimate time-varying reproduction numbers during epidemics. Am. J. Epidemiol..

[46] Fraser C. (2007). Estimating individual and household reproduction numbers in an emerging epidemic. PLoS ONE.

[47] Xu C., Dong Y., Yu X., Wang H., Tsamlag L., Zhang S., Chang R., Wang Z., Yu Y., Long R. (2020). Estimation of reproduction numbers of COVID-19 in typical countries and epidemic trends under different prevention and control scenarios. Front. Med..

[48] Tariq A., Banda J.M., Skums P., Dahal S., Castillo-Garsow C., Espinoza B., Brizuela N.G., Saenz R.A., Kirpich A., Luo R. (2021). Transmission dynamics and forecasts of the COVID-19 pandemic in Mexico, March-December 2020. PLoS ONE.

[49] Patrikar S., Kotwal A., Bhatti V., Banerjee A., Chatterjee K., Kunte R., Tambe M. (2020). Incubation Period and Reproduction Number for novel coronavirus 2019 (COVID-19) infections in India. Asia Pac. J. Public Health.

[50] Marimuthu S., Joy M., Malavika B., Nadaraj A., Asirvatham E.S., Jeyaseelan L. (2021). Modelling of reproduction number for COVID-19 in India and high incidence states. Clin. Epidemiol. Glob. Health.

[51] Shim E., Tariq A., Choi W., Lee Y., Chowell G. (2020). Transmission potential and severity of COVID-19 in South Korea. Int. J. Infect. Dis..

[52] Tariq A., Lee Y., Roosa K., Blumberg S., Yan P., Ma S., Chowell G. (2020). Real-time monitoring the transmission potential of COVID-19 in Singapore, March 2020. BMC Med..

[53] WHO Regional Office for South East Asia (2021). COVID-19 Weekly Situation Report—Week 33.

[54] CDC Delta Variant: What We Know about the Science. https://www.cdc.gov/coronavirus/2019-ncov/variants/delta-variant.html.

[55] Poudel A. Highly Infectious New AY.1 Variant of Coronavirus Found in Infected People. https://kathmandupost.com/health/2021/06/21/highly-infectious-new-ay-1-variant-of-coronavirus-found-in-swab-samples-of-infected-people.

[56] WHO Nepal Story on COVID-19 Vaccine Deployment: A Good Start. https://www.who.int/about/accountability/results/who-results-report-2020-mtr/country-story/2020/nepal-story-on-covid-19-vaccine-deployment-a-good-start.

[57] 5 Million Doses of J&J Vaccine Arrive in Nepal. https://kathmandupost.com/health/2021/07/12/1-5-million-doses-of-j-j-vaccine-arrive-in-nepal.

[58] Country Analysis: Daily Cases and Deaths over Severity of Public Health and Social Measures (PHSM)-Nepal. https://experience.arcgis.com/experience/56d2642cb379485ebf78371e744b8c6a.

[59] Dramé M., Teguo M.T., Proye E., Hequet F., Hentzien M., Kanagaratnam L., Godaert L. (2020). Should RT-PCR be considered a gold standard in the diagnosis of Covid-19?. J. Med. Virol..

